# Supplementary figures and images for: Heparan sulfate promotes TRAIL-induced tumor cell apoptosis
Source: eLife. 2024 Jan 24;12:RP90192. doi: 10.7554/eLife.90192 (PMC10945736; doi:10.7554/eLife.90192)

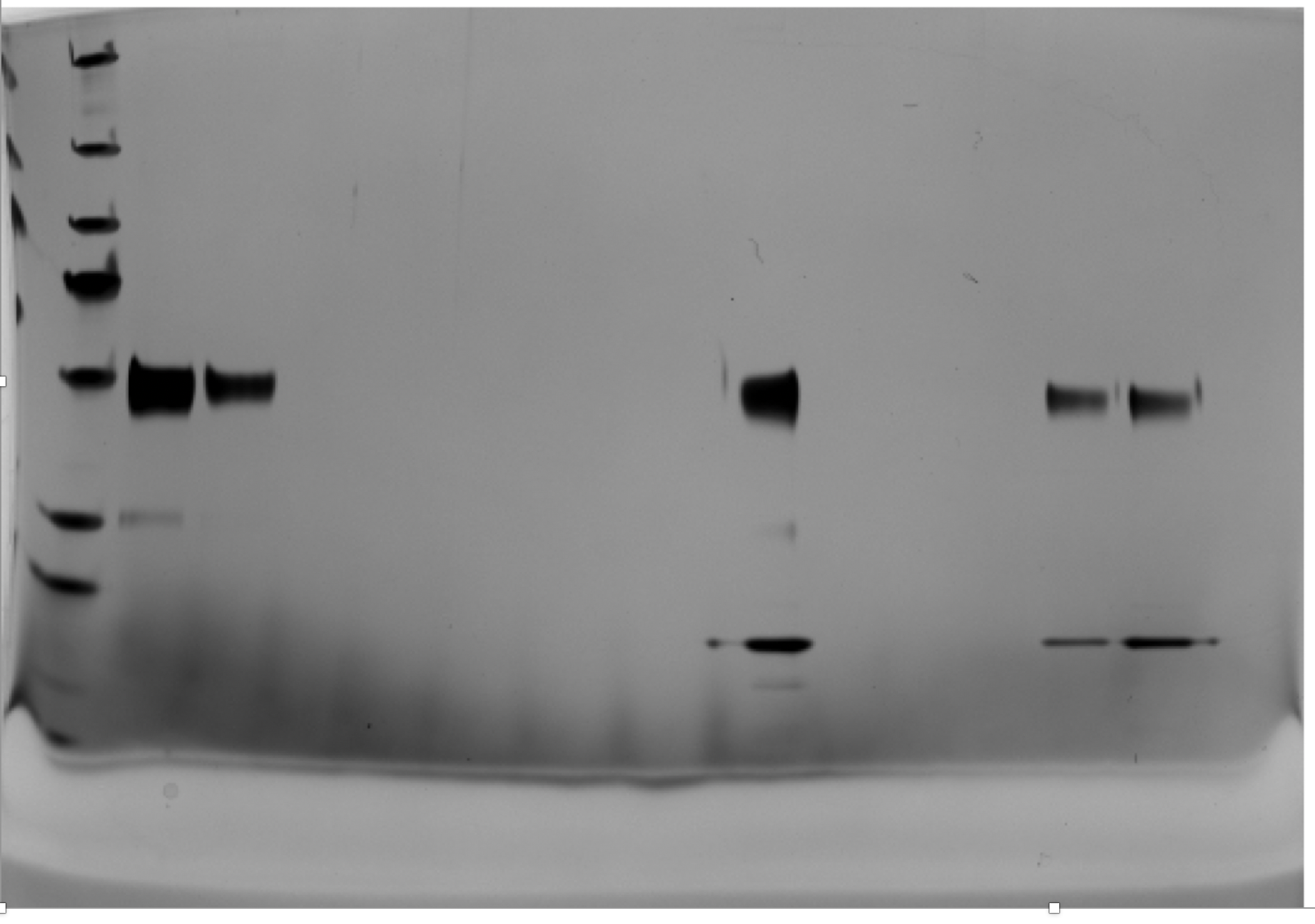

Supplement: Figure 9—source data 1. [file elife-90192-fig9-data1.zip › Figaure 9 source data 1.png]

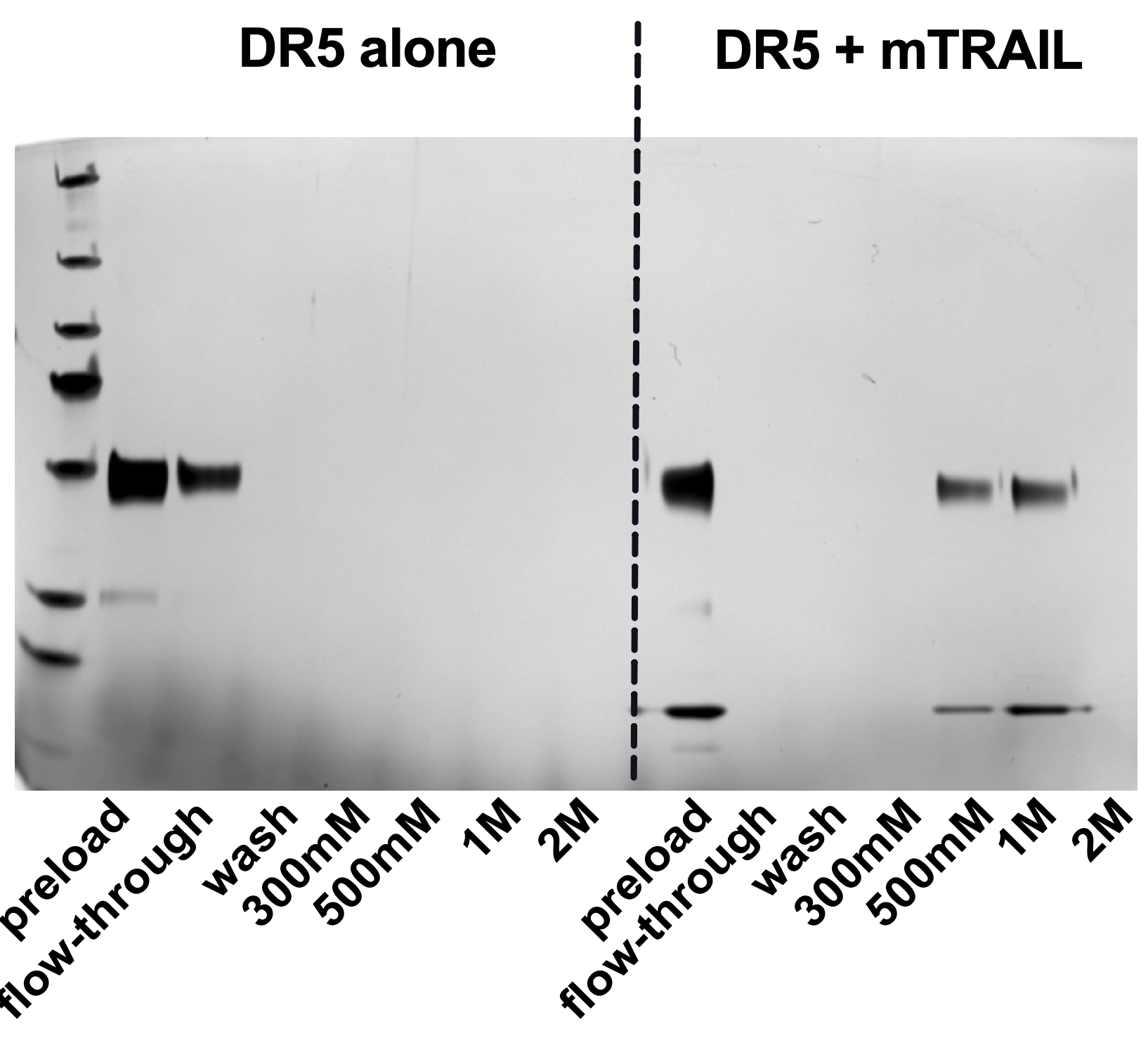

Supplement: Figure 9—source data 2. [file elife-90192-fig9-data2.zip › Figaure 9 source data 2.png]
